# The role of routine postoperative laboratory tests following hip hemiarthroplasty for an elderly femoral neck fracture

**DOI:** 10.1186/s12891-021-04698-4

**Published:** 2021-09-18

**Authors:** Teng-Feng Zhuang, Song-Wei Huan, Si-Min Luo, Guo-Rong She, Wen-Rui Wu, Jun-Yuan Chen, Ning Liu, Zhen-Gang Zha

**Affiliations:** The First Clinical College, Jinan University & Department of Orthopedics, The First Affiliated Hospital, Jinan University, Guangzhou, 510630 China

**Keywords:** Elderly, Femoral neck fracture, Hip hemiarthroplasty, Postoperative laboratory tests

## Abstract

**Background:**

Performing postoperative laboratory tests following joint arthroplasty is a regular practice. However, the role of routine postoperative laboratory tests in primary hip arthroplasty is currently in doubt. This study aimed to assess the role of routine postoperative laboratory tests for femoral neck fractures in elderly patients who underwent hip hemiarthroplasty and to evaluate the risk factors for postoperative laboratory testing abnormalities and related interventions.

**Methods:**

This retrospective study reviewed 735 consecutive patients with femoral neck fractures (FNFs) who underwent hip hemiarthroplasty at a single tertiary academic organization. Patient characteristic features and laboratory testing values were recorded. Logistic regression models were calculated to identify risk factors.

**Results:**

A total of 321 elderly patients (> 75 years of age) were ultimately enrolled for analysis. Abnormal postoperative laboratory tests were found in 265 patients (82.6%). Only a minority of the included patients (7.5%) needed medical intervention to treat postoperative laboratory testing abnormalities. Multivariate logistic regression analysis reported that a higher Charlson comorbidity index (CCI) (*P* = 0.03), abnormal preoperative haemoglobin level (*P* < 0.01), higher intraoperative blood loss (*P* < 0.01) and less frequent tranexamic acid use (*P* = 0.05) were risk factors for abnormal postoperative laboratory tests. Furthermore, a higher CCI has been identified as a risk factor for patients needing clinical interventions related to laboratory abnormalities.

**Conclusions:**

Because 92.5% of laboratory tests did not influence postoperative management, the authors suggest that routine laboratory tests after hip hemiarthroplasty for FNFs are less instructive for the majority of elderly patients. Nevertheless, for patients with identified risk factors, postoperative laboratory tests are still required to identify the abnormalities that need to be managed.

## Background

In the elderly population, the occurrence of hip fracture has been increasing rapidly due to the ageing of the population [1, 2]. With cautious estimations based on existing data, the prevalence of hip fractures could rise to 21.3 million in 2050. Furthermore, the main alterations will occur in Asian countries, where the percentage of hip fracture is projected to increase from 26% in 1990 to 45% in 2050 [3]. Displaced fractures (Garden type III and IV) comprise the majority of hip fractures [4, 5]. Hip hemiarthroplasty is considered one of the most effective orthopaedic surgeries to aim for pain relief and early mobilization in patients with displaced femoral neck fractures [6].

Surgeons are accustomed to performing postoperative laboratory tests routinely after joint replacement without evidence as to whether they are necessary. However, previous studies provided evidence that the application of routine postoperative laboratory tests was not required in patients with joint arthroplasty unless patients were at risk for laboratory testing abnormalities [7–9]. With the development of enhanced recovery after surgery (ERAS) in the field of joint replacement, vast advancements have been obtained in hip hemiarthroplasty in terms of both operative techniques and perioperative management [10]. To date, little is known about whether routine laboratory tests following hip hemiarthroplasty contribute to actionable information in elderly patients with displaced femoral neck fractures.

Thus, this trial aimed to report the frequency of abnormal postoperative laboratory tests, evaluate the incidence of interventions associated with laboratory abnormalities, and determine the risk factors related to abnormal laboratory results and interventions for laboratory abnormalities in patients who underwent hip hemiarthroplasty.

## Methods

This retrospective cohort study was performed with the approval of the ethics committee of the institute (The First Affiliated Hospital of Jinan University Institutional Review Board Approval Form No. KY-2020-034). Informed consent to participate in our study was obtained from all included patients. All participants who received hip hemiarthroplasty for displaced femoral neck fracture (Garden type III and Garden type IV) by the senior surgeon from January 2016 to January 2020 were included in the study. The inclusion criteria were as follows: age beyond 75 years at the time of the injury, mobility capacity without any aids prior to the fracture, and hip hemiarthroplasty treatment for displaced femoral neck fracture. Hip resurfacing arthroplasty or total hip arthroplasty (THA), pathologic fractures, previous history of hip surgery, and THA revision were excluded.

We collected the following data from the electronic medical records: age, sex, body mass index (BMI), American Society of Anesthesia (ASA) scores, Charlson comorbidity index (CCI), time from fracture to operation, operation time, intraoperative blood loss, tranexamic acid use, drainage tube use, preoperative and postoperative laboratory values, interventions that were related to postoperative laboratory test abnormalities, length of hospital stay, and 90-day emergency department (ED) visits or readmissions. Patient demographics are presented in detail in Table 1. The retrieved laboratory values are as follow: a complete blood count (haemoglobin) and a comprehensive metabolic panel (albumin, sodium, potassium, and calcium). We recorded laboratory results prior to the operation and on postoperative day 1. Information was collected from all patients after acquiring written informed consent in accordance with the Declaration of Helsinki. Normal laboratory values were defined as follows: haemoglobin (130–175 g/L in males and 115–150 g/L in females), albumin (35–50 g/L), serum sodium (137–147 mmol/L), serum potassium (3.5–5.3 mmol/L), and serum calcium (2–2.8 mmol/L). Abnormal laboratory test-related interventions included allogeneic red blood cell transfusion when the haemoglobin level was < 70 g/L, albumin supplementation when the albumin level was < 30 g/L, and electrolyte correction when electrolyte values were outside of normal defined ranges. Once laboratory results were determined to be abnormal, interventions were performed preoperatively or postoperatively.

**Table 1:** Demographic data and operative details for patients undergoing hip hemiarthroplasty

Variables	Normal Postoperative Laboratory Test (*N* = 56)	Abnormal Postoperative Laboratory Test (*N* = 265)	*P* value
**Preoperative variables**			
Age	82.07 ± 4.61	81.78 ± 5.73	0.80
Sex	39(F):17(M)	189(F):76(M)	0.80
BMI	24.51 ± 5.62	23.97 ± 5.42	0.14
ASA	2.25 ± 0.85	2.45 ± 0.88	0.1
CCI	3.03 ± 0.94	3.31 ± 1.27	0.01*
Time from fracture to operation	2.42 ± 1.04	2.55 ± 1.13	0.71
Hemoglobin	132.98 ± 8.07	113.89 ± 12.55	< 0.001*
Albumin	40.18 ± 3.54	32.06 ± 3.53	< 0.001*
Serum sodium	144.65 ± 3.36	145.58 ± 5.27	0.16
Serum potassium	4.27 ± 0.41	4.32 ± 0.67	0.77
Serum calcium	2.29 ± 0.13	2.26 ± 0.21	0.26
**Intraoperative variables**			
Operation time	54.84 ± 7.88	54.41 ± 8.01	0.75
Blood loss	95.25 ± 22.99	117.26 ± 31.2	< 0.001*
Tranexamic acid use	49(Yes):7(No)	126(Yes):139(No)	< 0.001*
Drainage tube use	14(Yes):42(No)	64(Yes):201(No)	0.89
**Postoperative variables**			
Hemoglobin	125.72 ± 6.53	104.42 ± 10.49	< 0.001*
Albumin	32.53 ± 3.88	29.71 ± 4.52	< 0.001*
Serum sodium	142.11 ± 5.45	141.56 ± 6.11	0.14
Serum potassium	3.7 ± 0.73	3.62 ± 0.84	0.35
Serum calcium	2.28 ± 0.12	2.26 ± 0.23	0.96
**Postoperative events**			
With intervention	0	24	0.02*
Transfusion	0	7	
Intravenous infusion of albumin	0	14	
Intervention for electrolytic disorders	0	3	
Length of hospital stay	8.4 ± 3.12	8.91 ± 3.44	0.20
90-day ED Visits or Readmissions	1	4	0.87

*BMI* body mass index, *ASA* American Society of Anesthesiologists, *CCI* Charlson Comorbidity Index, *ED* emergency department

All surgeries were performed by the senior surgeon with the patient under spinal anaesthesia. We performed hip hemiarthroplasty with a posterolateral approach using a bipolar hip prosthesis. The utility of tranexamic acid is part of the routine perioperative care unless the contradictions are present. Participants who were on a regimen of tranexamic acid were administered 1 g intravenously (IV) after the anesthesia and another 1 g IV during wound closure. All patients were managed under the enhanced recovery after surgery (ERAS) pathway. All participants allowed full weight bearing on postoperative day 1.

### Statistical analysis

The numbers of cases (n) and frequencies (percentages) were calculated for categorical variables, and the means ± standard deviations (SDs) or medians were calculated for continuous variables. Student’s t-test and chi-square test were used to analyse the differences between two groups in continuous variables and categorical variables, respectively. Univariate and multivariable logistic regressions were calculated to analyse the risk factors that were associated with postoperative laboratory test abnormalities and interventions for abnormal laboratory tests. We defined a *P* value < 0.05 as statistically significant. All statistical analyses were performed using R (R software, version 4.0.0.).

## Results

A total of 735 patients were evaluated for potential inclusion. Sixty-five patients who had a history of prior hip surgery were excluded (37 patients had been accepted hip arthroscopy, 28 patients had been accepted internal fixation for intertrochanteric fracture). Of those, 179 patients were excluded for missing or incomplete preoperative laboratory data (97 patients for missing complete blood count, 45 patients for missing comprehensive metabolic panel, and 37 patients for both), and 170 patients were excluded for missing or incomplete postoperative laboratory data (78 patients for missing complete blood count, 83 patients for missing comprehensive metabolic panel, and 9 patients for both). Ultimately, 321 patients were enrolled for analysis. The baseline demographic characteristics and intraoperative and postoperative data of the included patients with or without abnormal postoperative laboratory tests are shown in Table 1. A total of 265 patients who had either an abnormal complete blood count or an abnormal comprehensive metabolic panel were included in the abnormal postoperative laboratory test group, and the remaining patients who had normal postoperative laboratory tests were included in another group. The postoperative variables between the two groups were statistically comparable except for the CCI, haemoglobin level and albumin level. There was no transfusion before surgery. Three patients received albumin supplementation, and electrolytic abnormalities were corrected in only one patient preoperatively. In addition, no patients underwent albumin or blood transfusion during surgery. The group with postoperative laboratory abnormalities had a significantly greater CCI (3.03 ± 0.94 vs 3.31 ± 1.27, *P* = 0.01) and lower haemoglobin (132.98 ± 8.07 vs 113.89 ± 12.55, *P* < 0.001) and albumin levels (40.18 ± 3.54 vs 32.06 ± 3.53, *P* < 0.001). In addition, intraoperative blood loss (95.25 ± 22.99 vs 117.26 ± 31.2, *P* < 0.001) was significantly different between the two groups. The overall rate of tranexamic acid use in the normal postoperative laboratory test group was 87.5% (49 of 56), compared with 47.5% (126 of 265) in the abnormal postoperative laboratory test group. Statistically similar operation times (*P* = 0.75) and rates of drainage tube use (*P* = 0.89) were found in the two groups. All drainage tubes were removed 24 h after hip arthroplasty. The rates of clinical interventions were 0% (0 of 56) and 9.1% (24 of 265) in the groups with and without postoperative laboratory test abnormalities, respectively. The overall rate of clinical interventions for abnormal postoperative laboratory tests in the trial population was 7.5% (24 of 321). Hence, regular postoperative laboratory tests resulted in no revision of the postoperative procedure in 297 patients (92.5%). Furthermore, regular postoperative laboratory tests in the normal group were not related to a shorter length of hospital stay than in the group with abnormal laboratory tests (8.4 ± 3.12 vs 8.91 ± 3.44, *P* = 0.20). Ninety-day ED visits and readmissions were combined into one measurement due to their low prevalence. Mean 90-day ED visits or readmissions were not significantly different between the groups with or without postoperative normal laboratory tests (*P* = 0.87).

For patients with postoperative laboratory test abnormalities, there was a statistical univariate association with older age (*P* = 0.05), higher CCI (*P* = 0.01), lower preoperative haemoglobin (*P* < 0.01), higher intraoperative blood loss (*P* < 0.01) and less frequent tranexamic acid use (*P* = 0.04). Nevertheless, in the multivariate logistic regression model, significant predictors were the CCI (*P* = 0.03), preoperative haemoglobin level (*P* < 0.01), intraoperative blood loss (*P* < 0.01) and tranexamic acid use (*P* = 0.05), but not age (*P* = 0.08) (Table 2). Univariate analysis revealed a significant association between the CCI and intervention for laboratory test abnormalities (*P* = 0.01). Even though there was a trend towards significance with greater intraoperative blood loss, it was not statistically significant (*P* = 0.06). However, in the multivariable logistic regression analysis, the only variable that remained significant for predicting the risk of abnormal laboratory tests with intervention was the CCI (*P* = 0.04) (Table 3).

**Table 2:** Univariate and multivariable logistic regression analysis to identify risk factors for abnormal postoperative laboratory tests

Variables	Unadjusted odds ratio (95% CI)	*P* value	Adjusted odds ratio (95% CI)	*P* value
Age	1.13 (1.02 ~ 1.26)	0.05*	1.12 (0.99 ~ 1.26)	0.08
Sex	0.83 (0.77 ~ 1.18)	0.19	0.87 (0.77 ~ 1.09)	0.28
BMI	0.94 (0.81 ~ 1.08)	0.64	0.98 (0.89 ~ 1.07)	0.72
ASA	0.67 (0.33 ~ 1.24)	0.21	0.67 (0.34 ~ 1.25)	0.22
CCI	1.29 (1.17 ~ 1.41)	0.01*	1.28 (1.19 ~ 1.4)	0.03*
Time from fracture to operation	0.65 (0.39 ~ 1.09)	0.16	0.69 (0.39 ~ 1.08)	0.18
Preoperative hemoglobin	0.71 (0.39 ~ 0.97)	< 0.01*	0.75 0.42 ~ 0.98)	< 0.01*
Preoperative albumin	1.04 (0.83 ~ 1.25)	0.66	1.04 (0.89 ~ 1.22)	0.63
Preoperative serum sodium	0.69 (0.32 ~ 1.74)	0.39	0.67 (0.25 ~ 1.8)	0.41
Preoperative serum potassium	0.51 (0.3 ~ 1.17)	0.22	0.55 (0.34 ~ 1.23)	0.23
Preoperative serum calcium	0.37 (0.26 ~ 1.05)	0.17	0.36 (0.19–1.07)	0.18
Operation time	0.96 (0.93 ~ 1.02)	0.58	0.98 (0.92 ~ 1.04)	0.62
Blood loss	1.13 (1.02 ~ 1.38)	< 0.01*	1.09 (1.02 ~ 1.35)	< 0.01*
Tranexamic acid use	0.52 (0.31 ~ 0.98)	0.04*	0.54 (0.33 ~ 0.99)	0.05*
Drainage tube use	0.99 (0.55 ~ 1.23)	0.24	1 (0.6 ~ 1.21)	0.26

*BMI* body mass index, *ASA* American Society of Anesthesiologists, *CCI* Charlson Comorbidity Index, *ED* emergency department

**Table 3:** Univariate and multivariable logistic regression analysis to identify risk factors for abnormal postoperative laboratory tests with intervention

Variables	Unadjusted odds ratio (95% CI)	*P* value	Adjusted odds ratio (95% CI)	*P* value
Age	1.07 (0.97 ~ 1.19)	0.44	1.04 (0.93 ~ 1.13)	0.50
Sex	0.28 (0.19 ~ 1.07)	0.94	0.32 (0.21 ~ 1.14)	0.98
BMI	1.02 (0.93 ~ 1.11)	0.71	1.01 (0.9 ~ 1.14)	0.85
ASA	0.59 (0.31 ~ 1.09)	0.23	0.67 (0.34 ~ 1.27)	0.23
CCI	1.09 (1.03 ~ 1.15)	0.01*	1.05 (1.03 ~ 1.09)	0.04*
Time from fracture to operation	0.56 (0.24 ~ 1.27)	0.88	0.52 (0.23 ~ 1.24)	0.85
Preoperative hemoglobin	1.01 (0.96 ~ 1.05)	0.11	1.03 (0.98 ~ 1.09)	0.16
Preoperative albumin	0.98. (0.89 ~ 1.21)	0.26	1.06 (0.91 ~ 1.25)	0.40
Preoperative serum sodium	0.66 (0.25 ~ 1.24)	0.69	0.63 (0.29 ~ 1.17)	0.68
Preoperative serum potassium	0.77 (0.69 ~ 1.28)	0.44	0.71 (0.59 ~ 1.21)	0.44
Preoperative serum calcium	0.44 (0.38 ~ 1.09)	0.68	0.43 (0.38 ~ 1.05)	0.68
Operation time	0.99 (0.92 ~ 1.06)	0.11	0.93 (0.86 ~ 1.01)	0.12
Blood loss	0.94 (0.88 ~ 1.01)	0.06	0.98 (0.96 ~ 1.06)	0.19
Tranexamic acid use	1.28 (0.56 ~ 1.53)	0.66	1.31 (0.76 ~ 1.62)	0.69
Drainage tube use	0.73 (0.51 ~ 1.44)	0.85	0.76 (0.54 ~ 1.45)	0.99

*BMI* body mass index, *ASA* American Society of Anesthesiologists, *CCI* Charlson Comorbidity Index, *ED* emergency department

## Discussions

To our knowledge, there are no standardized routine laboratory test guidelines for fractures. The study of Alejandro et al. [11] was conducted using data from Spanish institute, and the routine laboratory test for hip fracture in elderly consisted of metabolic parameters and haematological parameters in the study. Hamid et al. [12] conducted a study in Iran and reported routine laboratory tests for hip fracture including, haemoglobin, sodium, potassium, blood urea nitrogen, and creatinine. Lee [13] et al. demonstrated that routine testing regrading albumin level was important for detecting poor nutritional status in order to reducing complications. However, as perioperative management has evolved, some authors suggested that less routine laboratory tests may be needed [14–16]. No evidence-based data evaluate the role of laboratory test for geriatric femoral neck fractures undergoing hemiarthroplasty. Therefore, this study aimed to comprehensively understand whether postoperative laboratory testing is instructive following hip hemiarthroplasty in elderly patients with displaced femoral neck fractures and to identify the risk factors using a logistic regression model.

To the best of our knowledge, the present study is the first to assess the role of routine postoperative laboratory tests in the scope of hip hemiarthroplasty. The major discovery of this study is that most patients (up to 82.6%) showed postoperative laboratory abnormalities, and only 7.5% (24 of 321) of patients needed to be treated with clinical interventions for abnormal laboratory results, which implied that regular postoperative laboratory testing did not contribute to actionable information. Of note, 14 of 24 patients underwent albumin supplementation due to hypoalbuminemia. Seven transfusions were carried out postoperatively based on haemoglobin levels below a certain threshold. However, the incidence of intervention for electrolytic disorders was very low. A higher CCI, abnormal preoperative haemoglobin, higher blood loss, and less frequent usage of tranexamic acid were verified as risk factors for postoperative laboratory testing abnormalities via both univariable and multivariable logistic regressions; however, older age was statistically significant only in the univariable logistic regression. Both unadjusted and adjusted odds ratios indicated that the CCI was a significant factor for predicting the utility of interventions for abnormal postoperative laboratory tests.

Femoral neck fractures are considered a significant public health problem because of both frequent incidence and severe impairment [17]. Hip hemiarthroplasty has been the most common selection for elderly femoral neck fractures and is believed to be a rapid and minimal blood loss procedure [18]. Historically, joint arthroplasty was recognized as a complex technique with a high frequency of transfusion [19]. Nevertheless, ERAS has obviously decreased the rate of transfusion to between 2 and 19% in the realm of joint replacement, and the present study demonstrated that the overall frequency of transfusion was 2.2% in patients undergoing hip hemiarthroplasty [20, 21]. Our centre developed an ERAS programme for hip arthroplasty based on the recommendation of Auyong et al. [22], including preoperative, intraoperative and postoperative components. The ERAS team consisted of experienced surgeons, anaesthetists, nursing teams, physical therapists, and nutritionists. For example, nutritionists are in charge of evaluating the patient’s nutritional condition and correcting nutritional deficiencies, such as hypoalbuminemia and nutritional anaemia. Previous studies have reported that the utility of postoperative laboratory testing was both unnecessary and cost-inefficient following joint arthroplasty [7, 23–25]. The study by Wu et al. [25] included that the incidences of abnormal postoperative laboratory results and interventions associated with abnormal values after THA were 88.4 and 6.8%, respectively, which were similar to the findings in the present study. Halawi et al. [26] showed that routine postoperative laboratory tests were performed without evidence in modern-day total hip arthroplasty and advised that laboratory tests should be performed selectively based on patients’ risk factors.

In this study, we found that the risk factors for postoperative laboratory testing abnormalities included greater CCI, abnormal preoperative haemoglobin level, higher blood loss, and less frequent usage of tranexamic acid. These results were consistent with prior studies. Kildow et al. [24] demonstrated that there was no evidence that actionable information could be obtained from patients without severe comorbidities based on a routine postoperative basic metabolic panel unless patients were diagnosed with diabetes, chronic kidney disease, or preoperative laboratory abnormalities. Wu et al. [27] conducted a study of THA for treating patients with hip fracture and reported a high occurrence of abnormal postoperative laboratory tests and a significant clinical intervention rate. Their results indicated that the utility of routine postoperative laboratory tests following THA is necessary for patients with long operative times and preoperative abnormalities. Compared to Wu et al. [27], the results of the present study indicated that a higher Charlson comorbidity index was a significant risk factor for predicting clinical intervention for postoperative abnormalities. Our findings were partially consistent with Halawi et al. [9], who reported that abnormal preoperative laboratory electrolyte levels and anaemia were indicators of abnormal postoperative laboratory results in total knee arthroplasty. The authors explored whether greater American Society of Anaesthesiologists scores, higher blood loss and lack of tranexamic acid use were risk factors for blood transfusion. However, in contrast to Halawi et al., our results found that a higher CCI was the only risk factor for treatments, including blood transfusion, intravenous infusion of albumin and intervention for electrolytic disorders, in elderly patients with femoral neck fractures undergoing hip hemiarthroplasty.

The Charlson comorbidity index (CCI), which was established in 1987, has been developed as a prospective factor for identifying comorbid conditions [28]. The Charlson index value is a total of 19 preset comorbid conditions that have distributed weights of 1, 2, 3, or 6 [28]. Our study found that a higher CCI was a strong predictor of both abnormal postoperative laboratory testing values and interventions related to abnormal values. This finding remained statistically significant even following multivariable adjustment for other factors of interest. The CCI has been used to predict the risk of early mortality in patients undergoing total hip arthroplasty for femoral neck fractures, and the results showed that a CCI > 3 was related to a dramatic increase in the risk of cardiovascular death within 90 days [29]. Jurisson et al. [30] retrospectively reviewed 8298 patients with incident femoral neck fractures and reported that the presence of preinjury comorbid conditions raised the risk of excess death in hip fracture patients. Jordan et al. [7] showed that 87.3% of postoperative laboratory tests did not lead to alterations in postoperative treatment, and a lower BMI, higher CCI and chronic kidney disease were proven to be risk factors for postoperative laboratory abnormalities via univariable analysis.

In the current study, we emphasized the critical significance of perioperative management procedures based on enhanced recovery after surgery protocols. On the one hand, preoperative laboratory abnormalities were treated as soon as the results were found to be abnormal; however, the abnormalities of some geriatric patients may have not been completely adjusted because of their poor general condition. Once laboratory results were found to be abnormal, interventions were performed preoperatively or postoperatively. In our results, 7.5% (24 of 321) of patients required clinical correction directly related to abnormal values after surgery. Previous studies [7, 9, 25] also reported that the incidence of related interventions was low, which was consistent with our findings. On the other hand, the majority of patients (*n* = 35) were administered tranexamic acid (TXA), which has the capacity to inhibit the conversion of plasminogen activation and delay fibrinolysis [31]. The utility of TXA has been successful in decreasing blood loss in orthopaedic surgery [32]. Lee et al. [33] retrospectively evaluated 271 patients with hip hemiarthroplasty and reported that the transfusion rate of the TXA group was significantly lower than that of the no-TXA group (6% vs 19%). Kang et al. [34] demonstrated that topical usage of TXA was a safe and effective solution in hip hemiarthroplasty, obviously reducing the postoperative drainage amount. Kwak et al. [35] indicated that TXA is believed to be a simple and helpful method for reducing bleeding, the transfusion rate and complications in geriatric femoral neck fractures after hemiarthroplasty. Our finding was consistent with the above studies. The absence of TXA use was another risk factor for abnormal postoperative laboratory testing, and the percentage of TXA use was significantly higher in the normal postoperative laboratory test group.

Subsequently, as with improvements in perioperative management and surgical techniques, the frequency of abnormal postoperative laboratory tests is less common in the majority of femoral neck fractures after hip hemiarthroplasty. However, for patients with a greater CCI, abnormal preoperative haemoglobin, higher blood loss, and less frequent usage of tranexamic acid, laboratory testing was warranted to identify potential abnormalities after surgery.

The present study has several limitations. First, due to the nature of the target population, it was difficult to eliminate heterogeneity entirely between the two groups. Second, this study was carried out in a single tertiary academic centre. Nevertheless, as a general care organization, we enrolled a great diversity of patients, resulting in widely practicable conclusions. Third, as a retrospective study, the nature of the study design was unable to accurately identify whether the postoperative laboratory testing abnormalities were directly related to any manifestations. And we did not assess the C reactive protein and leucocytes due to unavailable data regarding infection. Finally, bias may be present in the data for 90-day ED visits and hospital readmissions if the included patients sought medical help in other care centres, which may have led to an underestimation of the real frequencies of 90-day ED visits and readmissions.

## Conclusion

To conclude, majority of routine postoperative laboratory tests in elderly FNFs following hip hemiarthroplasty seldomly indicated actionable information. Higher CCI, abnormal preoperative haemoglobin level, higher intraoperative blood loss, and less frequent tranexamic acid use were risk factors for patients with abnormal postoperative laboratory results. With respect to risk factor for interventions with postoperative laboratory abnormalites, CCI was the only statistically significant finding. The current study adds to the growing evidence that postoperative laboratory testing abnormality is less frequent for majority of patients, especially for the elderly in present-day perioperative procedure. However, if the patient was in high risk, postoperative laboratory tests is still required to identify the abnormalities needed to be managed.

## Data Availability

The datasets supporting the conclusions of this article are included within the article. Raw data can be requested from the corresponding author on reasonable request.

## Abbreviations

FNF: Femoral neck fracture
CCI: Charlson comorbidity index
ERAS: Enhanced recovery after surgery
THA: Total hip arthroplasty
BMI: Body mass index
ASA: American Society of Anesthesia scores
CBC: Complete blood count
CPM: Comprehensive metabolic panel
SD: Standard deviation
TXA: Tranexamic acid
